# One-Step In Vitro Generation of ETV2-Null Pig Embryos

**DOI:** 10.3390/ani12141829

**Published:** 2022-07-18

**Authors:** Marta Moya-Jódar, Giulia Coppiello, Juan Roberto Rodríguez-Madoz, Gloria Abizanda, Paula Barlabé, Amaia Vilas-Zornoza, Asier Ullate-Agote, Chiara Luongo, Ernesto Rodríguez-Tobón, Sergio Navarro-Serna, Evelyne París-Oller, Maria Oficialdegui, Xonia Carvajal-Vergara, Laura Ordovás, Felipe Prósper, Francisco Alberto García-Vázquez, Xabier L. Aranguren

**Affiliations:** 1 Program of Regenerative Medicine, Centre for Applied Medical Research (CIMA), Instituto de Investigación Sanitaria de Navarra (IdiSNA), University of Navarra, 31008 Pamplona, Spain; mmoya.6@alumni.unav.es (M.M.-J.); gcoppiello@unav.es (G.C.); jrrodriguez@unav.es (J.R.R.-M.); gabizanda@unav.es (G.A.); pbarlabe@alumni.unav.es (P.B.); aullatea@unav.es (A.U.-A.); xcarvajal@unav.es (X.C.-V.); fprosper@unav.es (F.P.); 2 Advanced Genomics Laboratory, Program of Hemato-Oncology, Center for Applied Medical Research (CIMA), University of Navarra, 31008 Pamplona, Spain; avilaszo@unav.es; 3 Department of Physiology, Veterinary School, International Excellence Campus for Higher Education and Research (Campus Mare Nostrum), University of Murcia, 30100 Murcia, Spain; chiara.luongo@um.es (C.L.); rotoern@hotmail.com (E.R.-T.); sergio.navarro3@um.es (S.N.-S.); evelynemercedes.paris@um.es (E.P.-O.); 4 Institute for Biomedical Research of Murcia, IMIB-Arrixaca, 30100 Murcia, Spain; 5 Granja Los Alecos, 31395 Barásoain, Spain; maria@alecos.com; 6 Aragon Agency for Research and Development (ARAID), 50018 Zaragoza, Spain; lordovas@unizar.es; 7 Biomedical Signal Interpretation and Computational Simulation (BSICoS), Institute of Engineering Research (I3A), University of Zaragoza & Instituto de Investigación Sanitaria (IIS), 50018 Zaragoza, Spain; 8 Centro de Investigación Biomédica en Red de Cáncer (CIBERONC), 28029 Madrid, Spain; 9 Instituto de Investigación Sanitaria de Navarra (IdiSNA), 31008 Pamplona, Spain; 10 Department of Hematology and Cell Therapy, Clínica Universidad de Navarra, 31008 Pamplona, Spain

**Keywords:** gene editing, porcine embryos, CRISPR/Cas9, *ETV2*, vascular development

## Abstract

**Simple Summary:**

One of the latest goals in regenerative medicine is to use pluripotent stem cells to generate whole organs in vivo through the blastocyst complementation technique. This method consists of the microinjection of pluripotent stem cells into preimplantation embryos that have been genetically modified to ablate the development of a target organ. By taking advantage of the spatiotemporal clues present in the developing embryo, pluripotent stem cells are able to colonize the empty developmental niche and create the missing organ. Combining human pluripotent stem cells with genetically engineered pig embryos, it would be possible to obtain humanized organs that could be used for transplantation, and, therefore, solve the worldwide issue of insufficient availability of transplantable organs. As endothelial cells play a critical role in xenotransplantation rejection in all organs, in this study, we optimized a protocol to generate a vascular-disabled preimplantation pig embryo using the CRISPR/Cas9 system. This protocol could be used to generate avascular embryos for blastocyst complementation experiments and work towards the generation of rejection-free humanized organs in pigs.

**Abstract:**

Each year, tens of thousands of people worldwide die of end-stage organ failure due to the limited availability of organs for use in transplantation. To meet this clinical demand, one of the last frontiers of regenerative medicine is the generation of humanized organs in pigs from pluripotent stem cells (PSCs) via blastocyst complementation. For this, organ-disabled pig models are needed. As endothelial cells (ECs) play a critical role in xenotransplantation rejection in every organ, we aimed to produce hematoendothelial-disabled pig embryos targeting the master transcription factor *ETV2* via CRISPR-Cas9-mediated genome modification. In this study, we designed five different guide RNAs (gRNAs) against the DNA-binding domain of the porcine *ETV2* gene, which were tested on porcine fibroblasts in vitro. Four out of five guides showed cleavage capacity and, subsequently, these four guides were microinjected individually as ribonucleoprotein complexes (RNPs) into one-cell-stage porcine embryos. Next, we combined the two gRNAs that showed the highest targeting efficiency and microinjected them at higher concentrations. Under these conditions, we significantly improved the rate of biallelic mutation. Hence, here, we describe an efficient one-step method for the generation of hematoendothelial-disabled pig embryos via CRISPR-Cas9 microinjection in zygotes. This model could be used in experimentation related to the in vivo generation of humanized organs.

## 1. Introduction

The shortage of organs for use in transplantation is a recognized sanitary burden worldwide. To solve this problem, one of the latest goals in regenerative medicine is to produce humanized organs in farm animals via interspecies blastocyst complementation. This technique consists of the microinjection of PSCs in preimplantation embryos that have been genetically modified to be unable to form a given organ/tissue. In this environment, exogenous cells can colonize the empty developmental niche and contribute to the development of the targeted organ. Therefore, genetically modified host embryos impaired in the development of a targeted tissue need to be produced for these experiments. Furthermore, the disruption of genes that are crucial for organ development are useful not only in the application of blastocyst complementation for the generation of exogenous organs but also for the study of the mechanism of porcine organ development. The most common method used to obtain such models is the biallelic targeting of a key transcription factor (TF) determining cell lineage or organ development. These knockout (KO) embryos must be generated on a large scale, as the production of viable complemented animals interspecies is low, as demonstrated in rat-into-mouse experiments [1].

The domestic pig (*Sus scrofa*) is an optimal species for the generation of these humanized organs due to its anatomical and physiological similarities to humans, wide availability, short period of reproductive maturity and pregnancy (~4 months), and large number of offspring, in addition to the fact that there are fewer ethical concerns regarding its use compared to species that are more phylogenetically related to humans, such as primates [2].

Organ-disabled pig models such as the *PDX1* KO/mutated (pancreas-disabled) [3,4,5,6] and the *SALL1* KO (anephrogenic) [6,7] models have been generated, and some of them have been successfully complemented intraspecies using pig blastomeres, producing pancreases [3,6] and kidneys [6], respectively, the parenchymal cells of which were completely of exogenous origin, whereas the endothelial and stromal cells were formed from a mix of donor and host cells. However, ECs play a critical role in xenotransplantation rejection because they are enriched with epitopes and signaling molecules that trigger the immune response and the coagulation cascade [8]; therefore, the substitution of ECs in humanized organs is of pivotal importance, and to enable this, large-scale production of avascular pig embryos is a necessity. To date, two avascular pig models have been produced: the *KDR* KO [6] and the *ETV2* KO [9] models. They were both generated via somatic cell nuclear transfer (SCNT) using fibroblasts genetically modified with engineered endonucleases in vitro. Nonetheless, to perform SCNT, remarkable technical skills are required, and it is an extremely inefficient process, as only 1–2% of the cloned embryos produce viable offspring [10]. In addition, its use is associated with the abnormal epigenetic regulation of resultant offspring, which often present health complications and occasionally suffer from sudden death [11,12].

In recent years, CRISPR/Cas9 technology has emerged as a powerful tool for use in gene-edited pig production [13] as it delivers high gene-editing frequencies and can be used directly in pig zygotes, enabling one to circumvent the SCNT process. The CRISPR/Cas9 complex induces a DNA double-strand break of a target sequence. Site-directed mutagenesis is induced by the cell’s repair mechanisms by the generation of indels during the process of non-homologous end joining (NHEJ). If a donor template with homology arms is provided, a CRISPR/Cas9-induced double-strand break facilitates the insertion of foreign DNA. Additionally, entire genome fragments can be deleted by the concomitant introduction of two gRNAs [14]. Recently, several gene-edited pigs have been produced using this system [15,16,17], between them, three of the above mentioned *PDX1* KO [4,5] and *SALL1* KO [7] models. Nevertheless, this method must be optimized for each desired target gene. In the case of organ-disabled models for blastocyst complementation, efficiency optimization is especially important, as mutated embryos must be produced in high numbers for each experiment, and biallelic mutations are necessary to induce the expected phenotype.

In this study, we targeted the pig *ETV2* gene, which encodes for a TF belonging to the ETS TFs family and is expressed in hemangioblasts, controlling endothelial and hematopoietic cell fate. The deletion of *ETV2* produces embryos that are unable to generate endothelial and hematopoietic systems both in mouse [18] and pig [9]. Moreover, gene trapping before the DNA-binding domain has been shown to disrupt Etv2 function [19]. Therefore, in this study, we microinjected a CRISPR/Cas9 system in pig zygotes to disrupt *ETV2* by targeting its DNA-binding domain. Using a combination of two gRNAs, we were able to produce biallelic *ETV2*-mutated porcine blastocysts in an effective manner. 

## 2. Materials and Methods

### 2.1. Pig Fibroblast Isolation

Porcine fibroblasts were obtained from a skin biopsy of an adult female hybrid minipig. Briefly, skin was cut in small pieces and incubated in Collagenase I solution (Gibco; 1 mg/mL) in PBS (phosphate-buffered saline, Sigma-Aldrich, Madrid, Spain) at 37 °C for 1 h. After enzymatic digestion, skin pieces were mechanically disaggregated, filtered through a 40-µm cell strainer, washed with PBS, and centrifuged at 600 g for 6 min. Isolated cells were cultured on gelatin-precoated flasks (0.1% gelatin (Sigma-Aldrich) at 37 °C for at least 30 min. Culture media components: DMEM High Glucose, 15% fetal bovine serum (FBS), 1% Glutamax, 1% NEEA, 1% antibiotic/antimycotic solution, and 0.1 mM β-mercaptoethanol. The day after seeding, the medium was replaced to remove all non-attached cells. Every 3–4 days (80% confluence), cells were split using TrypLE™. All of the above-mentioned reagents were obtained from Gibco (Thermo Fisher Scientific, Madrid, Spain).

### 2.2. Guide RNA Design and In Vitro Testing

CRISPR RNAs (crRNAs) targeting the coding sequence of the DNA-binding domain of *ETV2* in exon 5 were designed using www.cripr.mit.edu/guides online resource (accessed on 1 July 2015). The selected 20-nt sequences upstream of the 5′-NGG-3′ PAM sequence of SpCas9 were: 5′ AGCTGCTACGCGTCCCGTCG3′ (crRNA1); 5′ GGTCGCACAGTTGGAACTCG3′ (crRNA2); 5′ CGCGTAGCAGCTGCATCCGC 3′ (crRNA3); 5′ CGCGGCTGTTGCCCGTCCAG3′ (crRNA4), and 5′ TCCTGGAGCTGCTCCACGAC 3′ (crRNA5). Annealed oligonucleotides (Thermo-Fisher Scientific) were cloned into the BbsI restriction site of the pSpCas9(BB)-2A-GFP (PX458) plasmid (Addgene #48138) using standard molecular cloning techniques. To test the guides’ targeting efficiencies, the cloned PX458 plasmids containing the CRISPR/Cas9 system were individually nucleoporated into cultured pig fibroblasts: 5 µg of plasmid was nucleoporated in 10^6^ cells using the Amaxa^®^ Human Dermal Fibroblast Nucleofector Kit (Lonza #: VPD-1001), giving 3 pulses of the program A24 with Amaxa nucleofector II (Amaxa Biosystems AAD-10015). Nucleofected fibroblasts were seeded and cultured for 72 h.

### 2.3. Surveyor Analysis

Fibroblast genomic DNA was extracted using a NucleoSpin tissue kit (Macherey-Nagel, Düren, Germany) and quantified using a NanoDrop. Indel detection with Surveyor nuclease digestion (Transgenomic) was conducted by following the manufacturer’s instructions. Briefly, the target region was amplified from 50 ng of gDNA via PCR with Platinum Taq enzyme (#11304-01, Invitrogen™, Barcelona, Spain) using the following primers: ETV2-Surveyor_F: 5′ CCTGGGTCATCGAAACGACA 3′ and ETV2-Surveyor_R: 5′ CCCGGCTTAGTCCGAGAG 3′ at a 200 nM concentration. PCR samples were denatured by being warmed to 95 °C for 5 min and were left to cool to room temperature to allow for self-hybridization. Hybridized samples were treated with Surveyor nuclease with an incubation of 60 min at 42 °C. Kit control heteroduplex–homoduplex samples were taken as internal controls. Non-nucleoporated pig fibroblasts were also taken as controls. Digested samples were run on 2% agarose gel with 0.1% ethidium bromide and visualized with a UV transilluminator (Bio-Rad, Madrid, Spain).

In order to identify the origin of the 200-bp band that appeared in all of the samples after surveyor digestion, we cloned the PCR product of the control samples from the primers ETV2-Surveyor_F: 5′ CCTGGGTCATCGAAACGACA 3′ and ETV2-Surveyor_R: 5′ CCCGGCTTAGTCCGAGAG 3′ into a TOPO-TA vector, transformed TOP10 competent bacteria (TOPO-TA cloning kit K450002 Thermo Fisher scientific) with this ligation product, and picked 9 colonies. Sequencing of the plasmid DNA derived from these clones was performed with M13 primer. 

### 2.4. Oocyte Collection and In Vitro Maturation (IVM)

Unless otherwise indicated, all of the chemicals and reagents were purchased from Sigma-Aldrich.

Ovaries were collected from prepubertal gilts (crossbred of Landrace and Large white) at a slaughterhouse (El Pozo S.A, Alhama de Murcia, Murcia, Spain) and transported in tempered (38.5 °C) physiological saline solution containing 1 g/L kanamycin sulfate. Upon arrival, the ovaries were washed with a 0.04% solution of cetyltrimethylammonium bromide (CTAB) and subsequently rinsed twice with saline solution, both previously tempered at 38.5 °C. Within 3 h after the sacrifice, the cumulus–oocyte complexes (COCs) were extracted via follicular punctures using 10 mL syringes and 19G needles. Subsequently, COCs with a homogeneous nucleus, ooplasm, and at least 3 compact layers of cumulus cells were selected and washed twice in Dulbecco’s PBS (DPBS) supplemented with 1 g/L polyvinyl alcohol (PVA) before IVM. The collected COCs were matured at 38.5 °C and 5% CO_2_ in groups of 50–55 in a 4-well plate (NUNC) with 500 µL of NCSU-37 medium [20] supplemented during the first 22 h with porcine follicular fluid, PMSG/hCG, and dAPMc, as previously described [21]. Then, COCs were transferred to NCSU-37 medium in the absence of hormones and dAMPc for an additional 22 h in the same culture conditions. Ninety percent of the initial immature oocytes achieved successful in vitro maturation, displaying chromosomes at Metaphase II (MII) and extrusion of the first polar body (PB). 

### 2.5. In Vitro Fertilization (IVF)

After the IVM of oocytes, IVF was carried out with fresh semen from fertile-tested Duroc boars (CEFU S.A., Murcia, Spain). Upon arrival at the laboratory, a basic semen analysis was performed, where the sperm concentration, motility, and viability were calculated using the CASA system utilizing the ISAS^®^ software (PROiSER R+D S.L., Valencia, Spain). Then, a spermatic selection was carried out by means of the Percoll^®^ gradient [22]. For this purpose, 2 mL of 90% Percoll^®^ solution was placed in a 15 mL conical tube, and subsequently, 2 mL of 45% Percoll^®^, made using a 1:1 dilution of 90% Percoll^®^: Beltsville thawing solution (BTS), was added. On top of the Percoll phases described above, 500 µL of fresh pure semen was deposited. The sample was centrifuged at 700× g for 30 min without brake at room temperature. The supernatant was discarded, and 10 mL of TALP medium (consisting of 114.06 mM NaCl, 3.2 mM KCl, 8 mM Ca-lactate.5H_2_O, 0.5 mM MgCl_2_.6H_2_O, 0.35 mM NaH_2_PO_4_, 25.07 mM NaHCO_3_, 1.85 mM Na-lactate, 0.11 mM Na-pyruvate, 5 mM glucose, 2 mM caffeine, 1 mg/mL PVA, and 0.17 mM kanamycin sulfate) was added to wash the spermatozoa pellet, previously supplemented with 3 mg/mL BSA and 0.11 mM sodium pyruvate and equilibrated at 38.5 °C, 5% CO_2_, and 5% O_2_ for at least 3 h before its use. Sperm was centrifuged at room temperature at 700× *g* for 10 min with brake. Next, the supernatant was discarded, and spermatozoa were resuspended in 1 mL of TALP medium. Afterwards, the sperm concentration was evaluated in a Neubauer chamber (VWR International, Haasrode, Belgium) using a dilution of 1:100 in a formalin saline solution. Sperm suspension was diluted to a final concentration of 20,000 spermatozoa/mL in TALP medium. Mature oocytes were prepared for IVF by mechanically removing cumulus cells via soft pipetting, and they were placed in a 4-well plate containing 250 μL of TALP medium in groups of 50–55 oocytes per well. Subsequently, 250 µL of diluted semen was added to each well containing denuded oocytes. Spermatozoa and oocytes were cocultured for 4 h at 38.5 °C and 5% CO_2_ and 5% O_2_. Hereinafter, putative zygotes were softly pipetted to remove excessive sperm that adhered to the zona pellucida and cultured for 2 h in TALP medium until CRISPR/Cas-9 microinjection. In order to determine the efficiency of the IVF, a representative number of these putative zygotes was treated with Hoechst 33,342 DNA staining, and the following parameters were evaluated under a fluorescence microscope (Leica^®^ DM4000 Led, Wetzlar, Germany, 460/490 nm): penetration rate (percentage of oocytes penetrated by at least one spermatozoon), monospermy rate (percentage of oocytes penetrated by only one spermatozoon from the total penetrated), and IVF efficiency rate (percentage of oocytes that were penetrated and monospermic from the total number inseminated). The penetration rate obtained was 70.1%, with 43.7% being monospermic, achieving a 30.7% IVF efficiency rate, demonstrating the optimal performance of this technique.

### 2.6. In Vivo Fertilization and Zygote Collection

Sows (crossbreeds of Landrace and Large white) were superovulated with 1000 IU PMSG and 72 h later with 750 IU hCG and inseminated 6–8 h after at the farm ‘Los Alecos’ (Barásoain, Navarra. Spain). The next day, presumably pregnant sows were transported to the animal facility at The University of Navarra. Preoperative anesthetic treatment consisted of a cocktail administered via the i.m. injection of 6 mg/kg tiletamina/zolazepam (Zoletil 100, Virbac, Barcelona, Spain) and 0.1 mg/kg medetomidine (Domitor, Pfizer S.A. Madrid, Spain). Anesthesia was maintained with isoflurane (Isoflo, Laboratorios Dr. Esteve S.A. Barcelona, Spain) vaporized in oxygen at 2–3% and fentanyl (Fentanest 0.05 mg/mL. Kern Pharma, Barcelona, Spain) in continuous infusion as an analgesic. The uteruses were surgically removed, and then, sows were euthanized with 3 mEq/kg i.v. potassium chloride (40 mEq/20 mL solution, B. Braun, Barcelona, Spain). Uteruses were transported in tempered (38.5 °C) physiological saline solution, containing 1 g/L kanamycin sulfate. Once in the lab, uteruses were perfused with TL-PVA-medium [23], and the collected zygotes were cultured in NCSU-23A [20,24] until CRISPR/Cas-9 microinjection.

### 2.7. Alt-R™ CRISPR/Cas9 Ribonucleoprotein Complex Formation

Cas9 protein, tracrRNA, and crRNA were purchased from Integrated DNA Technologies (IDT). Guide RNA/Cas9 RNP complex was formed by following the manufacturer’s instructions: for gRNA complex preparation, we resuspended crRNA and tracrRNA with an injection buffer (1 mM TrisHCl, pH = 7.5; 0.1 mM EDTA) to a final concentration of 1 µg/µL and mixed 5 µg of crRNA and 10 µg of tracrRNA, resulting in a final volume of 15 µL. Hereafter, annealing was performed in a thermocycler: 95 °C for 5 min, which was then ramped down to 25 °C at 5 °C/min. For the RNP complex formation, 5 µL (5 µg in total) of annealed gRNA complex was mixed with 1 µL of Alt-R S.p. Cas9 nuclease 3NLS at 10 µg/µL (10 µg in total) and diluted in 94 µL of injection buffer, obtaining a final volume of 100 µL. The mix was incubated at room temperature for 15 min to allow for the formation of RNP complexes in a solution containing the final concentration of 100 ng/µL Cas9 protein and 50 ng/µL gRNA complex. Before microinjection, the injection mix was centrifuged at 15,000× *g* for 10 min at room temperature. Supernatant (80 µL) was recovered and passed through a Millipore filter (UFC30VV25) in order to eliminate any solid particles and prevent clogging of the microinjection needles. For the preparation of the 5× (500 ng/µL Cas9 protein and 250 ng/µL gRNA complex) and 10× (1 µg/µL Cas9 protein and 500 ng/µL gRNA complex) microinjection mix, all of the above-mentioned quantities were scaled up proportionally.

### 2.8. Microinjection of CRISPR/Cas9 in Porcine Zygotes

RNP complex was microinjected into the in vitro and in vivo fertilized porcine zygotes. To perform the microinjection, porcine zygotes were transferred to 50-µL microdroplets of NCSU 23A medium overlaid with mineral oil and tempered at 38.5 °C, employing a thermoplate to maintain the temperature during microinjection. Using a CellTram vario microinjector (Eppendorf, Hamburg, Germany) or an Eppendorf TransferMan NK2 microinjector coupled to a Leica DMI 3000B microscope, individual zygotes were fixed via suction to a holding pipette (glass pipette with 120 µm of OD and a 35 °C angle from Vitrolife, #15306 or Eppendorf #5195 000.036) while the injection capillary (ICSI pipette with 4–5 µm of ID and a 30° angle from Vitrolife, #15444) was inserted into the zygote through the zona pellucida and the cell membrane, discharging RNP complex in each zygote’s cytoplasm. After the penetration of the zona pellucida and cytoplasm, successful injection was ensured by the visualization of the movement of the interface between the injection solution and the oil inside the microinjection capillary, without the accumulation of this volume between the zona pellucida and the plasma membrane. Injected zygotes were then transferred in groups of 40–45 into 500 µL of NCSU-23A medium covered with 300 µL of NidOil™ and cultured at 38.5 °C, 5% CO_2_, and 5% O_2_ in humidified air.

### 2.9. Embryo Culture

Microinjected and non-microinjected control embryos were cultured for 48 h in 500 µL of NCSU-23A porcine embryo culture medium, supplemented as previously described [24], and equilibrated at least 3 h before its use at 38.5 °C, 5% CO_2_, and 5% O_2_ in humidified air. Cleaved embryos featuring equal blastomeres and homogeneous cytoplasms were selected for further culture 48 h post microinjection while the non-divided embryos were discarded from both the microinjected and control groups. Thereafter, cleaved embryos were cultured in 500 µL of NCSU-23B [20] medium overlaid with 300 µL of NidOil™ for 96 h at 38.5 °C, 5% CO_2_, and 5% O_2_ in humidified air. The medium placed in a 4-well Nunc dish was equilibrated at least 3 h before its use.

### 2.10. Genomic DNA Extraction, Library Preparation for Next-Generation Sequencing (NGS), and Indel Detection

The blastocyst stage was evaluated 6 days after zygote microinjection. Embryos were individually collected in 10 µL of crude DNA extraction buffer (250 µg/mL proteinase K, 200 nM Tris-HCl (pH = 8.5), 200 nM KCl, 0.02% gelatin, and 0.45% Tween 20). For genomic DNA (gDNA) extraction, samples were placed in a thermoblock for 30 min at 56 °C, and then for an additional 10 min at 95 °C. The PCR amplification of *ETV2* was carried out in a 2720 Thermal Cycler (Applied Biosystem) with primers spanning the target site and having the following sequence: TAG-ETV2-Fwd: 5′ CACGACGCTCTTCCGATCTGTCCCGAGGAGGAGAGGATC 3′ and TAG-ETV2-Rev: 5′ CTGGAGTTCAGACGTGTGCTCTTCCGATCTGAGTTGAGATGAGGGCGAGG 3′. A KAPA HiFi Hot Start Ready Mix PCR Kit (#7958935001, Kapa Biosystems, Wilmington, United States) was used for DNA amplification. PCR products were purified using an Agencourt AMPure XP system (#A63881, Beckman Coulter, Barcelona, Spain) and quantified with a Qubit^®^ dsDNA HS Assay Kit (#Q32851, Invitrogen, Barcelona, Spain) in accordance with the manufacturer’s instructions. A second round of PCR was performed using 10 ng of the purified PCR products using universal primers with specific barcodes for each blastocyst to generate Illumina amplicons. These amplicons were purified as described above. The final library was made by mixing equal amounts of the second PCR products from all of the samples, which were then sequenced on an Illumina MiSeq (2 × 250 bp paired-end) at >25,000× coverage at amplified regions. Data were processed according to standard Illumina sequencing analysis procedures. Processed reads were aligned to the reference (Sscrofa11.1/susScr11) using the Burrows–Wheeler Aligner (BWA). Insertions and/or deletions were determined by comparing the reads against the reference sequence using a CrispRVariants R-based toolkit [25].

### 2.11. Statistical Analysis

The statistical analysis was performed using the GraphPad software. The chi square statistic test was used to determine significant differences in the cleavage and blastocyst rates between conditions (the embryo was considered as the experimental unit). Differences were considered statistically significant at *p* < 0.05.

## 3. Results

### 3.1. In Vitro Selection of Functional ETV2 Guide RNAs

**Figure 1:**
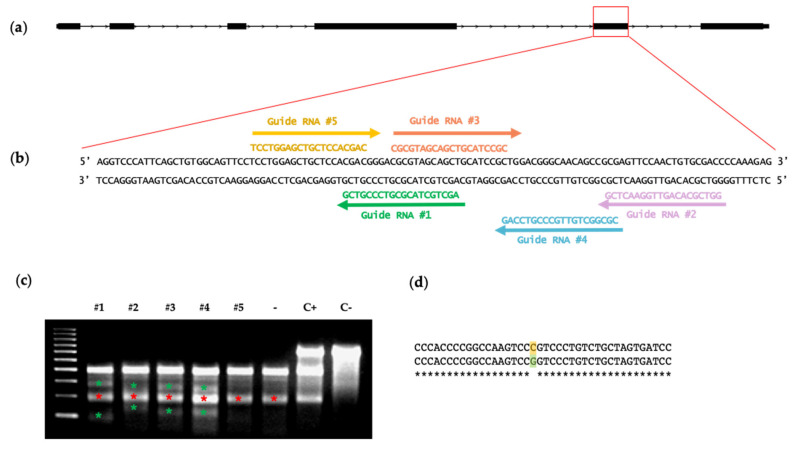
(**a**) Representation of the pig *ETV2* gene structure; The ETS-binding domain is encoded by exon 5 and 6. (**b**) The genomic target sequence and top five guides designed to target the *ETV2* exon 5; (**c**) Analysis of the Surveyor digestion showing that guide RNAs #1 to #4 are functional in vitro while guide 5 did not mediate the Cas9 activity; specific bands are highlighted with green asterisks. One band at 200 bp that does not correspond to CRISPR/Cas9 gene editing is present in all pig fibroblast samples, including the negative control (−); red asterisks. C+: kit’s GC heterodimer and C−: CC homodimer are included as positive and negative control of Surveyor activity, respectively; (**d**) an SNP in the genomic DNA of porcine fibroblasts was discovered by comparing the sequences of 9 different PCR samples from control fibroblasts. The SNP (C/G) is shown with colors. Its position on *ETV2* explains the 200-bp band produced by Surveyor analysis.

We designed crRNAs targeting the ETS DNA-binding domain of *ETV2* [26], specifically against exon 5, a key region for the TF protein function [27] (Figure 1a,b). We chose the top five crRNAs from the output (Figure 1b) and cloned them individually in PX458 plasmid, which also contained the tracrRNA and *S. pyogenes* Cas9 sequences, along with the GFP reporter gene, in order to test their targeting efficiency in vitro. Each plasmid was nucleoporated in cultured pig fibroblast. The efficiency of transfection estimated using the detection of the GFP reporter was ~50%. Indel generation was tested 72 h after nucleoporation via Surveyor analysis: gRNA 1 to 4 mediated on-target Cas9 double-strand break, resulting in indel production (Figure 1c, red asterisks) while gRNA 5 did not and, therefore, this guide was not used any further. Surveyor nuclease heteroduplex digestion also revealed the presence of an SNP in the fibroblast genomic DNA (Figure 1d), which was apparent as a band at 200 bp (Figure 1c, green asterisks) in all of the pig fibroblast samples.

### 3.2. Production of ETV2-Mutated Pig Blastocysts with Single gRNAs

**Figure 2:**
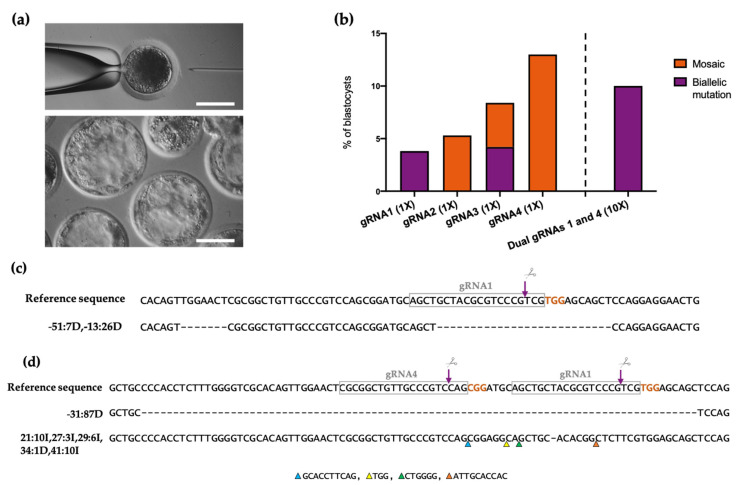
(**a**) Phase contrast microscope photography of a zygote during the process of CRISPR/Cas9 microinjection (upper panel) and microinjected embryos that reached the blastocyst stage upon in vitro culture (bottom panel). Scale bars: 100 μm; (**b**) bar graphs showing the percentage of blastocysts edited in the *ETV2* gene upon Cas9/gRNA microinjection (with individual or dual gRNAs). Orange bars indicate the percentage of mosaic gene editing and purple bars indicate the percentage of biallelic gene editing; (**c**) deletion obtained with gRNA1; (**d**) indels obtained with combined gRNA1 and gRNA4 microinjection at a 10X concentration. Gray rectangles indicate the guide homology regions. The PAM sequence is labeled in orange. The purple arrows mark the cleavage site position and corresponds to base 0.

The four functional gRNAs targeting the DNA-binding domain of *ETV2* were individually hybridized with Cas9 protein, obtaining RNP complexes with a concentration of 50 ng/μL of gRNA and 100 ng/μL of Cas9. These RNPs were individually microinjected into putative zygotes 6 h after in vitro fertilization (Figure 2a, upper panel). Microinjected and non-microinjected (control) zygotes were cultured for 6 days (Figure 2a, bottom panel). CRISPR/Cas9 complex microinjection at the concentration used did not negatively affect embryos’ development, as shown in the cleavage rate and blastocyst rate evaluation (Table 1). On day 6 post microinjection, a total of 120 blastocysts from all of the conditions (control, gRNAs 1 to 4) were collected and were individually analyzed for *ETV*2 gene disruption (Table 1). The region coding for the *ETV2* DNA-binding domain in exon 5 was amplified using two rounds of PCR, creating barcoded libraries that were analyzed using next-generation sequencing (NGS). Our results showed that mutant embryos were successfully produced using all of the microinjected guides (Figure 2b). In particular, gRNA1 and 3 allowed the generation of ~4% biallelic *ETV*2-mutated embryos (Figure 2b,c). It is noteworthy that gRNA1 did not generate any mosaic mutation while gRNA3 additionally produced ~4% of mosaic embryos. On the other hand, gRNA2 and 4 generated ~5% and 13% of edited embryos, respectively, with all of them being mosaic (Figure 2b).

### 3.3. Improvement of the Mutation Rate with Dual gRNAs

Next, we sought to combine the gRNA that gave the highest rate of KO generation (3.8%, gRNA1) with the gRNA that gave the highest rate of mutation (13%, gRNA4) in order to increase the editing efficiency of our system. Moreover, we increased the RNP components’ concentration to 5× (250 ng/μL gRNAs and 500 ng/μL Cas9) and 10× (500 ng/μL gRNAs and 1 μg/μL Cas9). CRISPR/Cas9 complexes were microinjected into zygotes obtained via in vivo fertilization, which were then cultured for 6 days. We observed that the cleavage rate was proportionally affected by increasing concentrations of RNP complexes (Table 2, *p* < 0.05). On the other hand, the blastocyst rate was not significantly decreased in microinjected embryos when compared to the control group (Table 2). On day 6 post CRISPR microinjection, we collected all of the blastocysts and analyzed them using NGS. In the 10× condition, it was determined that 10% of the embryos contained biallelic mutations (Figure 2b) while in the 5× condition, we did not detect any mutations (data not shown). These results indicate that by combining gRNAs 1 and 4 at 500 ng/μL and increasing the concentration of Cas9 protein to 1 μg/μL, we obtained the highest rate of biallelic mutation among the different conditions studied. The indels produced with combined gRNA1 and 4 at 500 ng/μL are represented in Figure 2d.

## 4. Discussion

Despite the outstanding potential of the blastocyst complementation approach to produce humanized organs in farm animals, several technical limitations need to be overcome to make its use a reality. The efficient generation of organ-disabled pig embryos is one of the challenges that should be addressed in order to optimize this technology. KO animals are traditionally obtained by generating heterozygous colony founders and their posterior breeding, especially when the KO phenotype is not compatible with life (as in the case of organ-disabled embryos) [1,28,29,30]. However, this approach is not viable for blastocyst complementation in pigs, as it would require the generation of a large colony of gene-edited pigs to serve as donors and would be highly costly and inefficient due to the long time required to complete their maturity development. On the other hand, engineered endonuclease-mediated gene-editing technologies developed in the last decade, such as the CRISPR/Cas9 system, have paved the way for the achievement of such a challenging objective as the large-scale direct generation of biallelic KO pig embryos in a one-step process [4,5,7]. For these reasons, it is preferable to introduce the desired biallelic mutation in wild-type embryos for every blastocyst complementation experiment. In this study, we assayed the generation of hematoendothelial-disabled pig embryos via CRISPR/Cas9-mediated gene editing in zygotes. We focused on the endothelium, as ECs play a critical role in organ rejection following pig-to-primate xenotransplantation [31,32]. First, we tested by zygote microinjection four functional gRNAs targeting the DNA-binding domain of the endothelial master gene *ETV2* in order to determine the gRNA that induced the highest mutation rates. gRNA1 induced the highest rate of biallelic mutation (~4%) while gRNA4 induced the highest rate of overall mutation (13% mosaic). After CRISPR/Cas9 microinjection, we obtained a blastocyst rate ranging from 19.15 to 60.47%, in line with the average progression to the blastocyst stage after IVF, which is approximately 30% according to the literature [33]. It is noticeable that two of the guides (gRNA1 and 2) provided higher blastocyst rates than the control (non-injected oocytes). There are several possible explanations that are not mutually exclusive: first, the oocyte injection per se may produce an increase in oocyte activation as it has been reported previously [34]; second, the number of zygotes used with these gRNAs was lower than the other groups, which may unbalance the final blastocyst rates; third, the used gRNA per se may affect embryo development, as some reports have pointed out previously [35]. Despite these differences, the number of blastocysts analyzed via NGS to evaluate the mutations was similar between groups (ranged from 19 to 28). In a second set of experiments, we combined gRNA1 and 4 and tested a higher concentration of RNP components: among the tested conditions, we determined that the best results were obtained by combining gRNA1 and gRNA4 at 500 ng/μL each and Cas9 protein at 1 μg/μL, which enabled the production of 10% biallelic mutated blastocysts. Several strategies were implemented to avoid the most common pitfalls associated with CRISPR/Cas9 technology. Firstly, we microinjected Cas9 protein instead of Cas9 mRNA, as delays in mRNA translation can increase mosaicism [36]. Secondly, we combined two different gRNAs to increase the generation of biallelic mutation, and finally, we titrated the concentration of RNP components in order to optimize the gene-editing efficiency without excessively compromising the embryo viability. 

The xenotransplantation of organs from hematoendothelial-disabled complemented pigs to primates will determine whether the substitution of merely the endothelial and hematopoietic compartments will be enough to enable successful pig-to-primate transplantation. If this is the case, this model would be of immense value as each and every vascularized organ could be retrieved from a single pig for xenotransplantation. On the contrary, if the substitution of more cell types is necessary to avoid transplant rejection, CRISPR/Cas9 gene targeting on zygotes could be used to ablate an additional gene, along with *ETV2* [37], to also hamper, for example, the parenchymal cell development of an organ of interest. A similar endeavor was undertaken by Matsunari et al., who generated a combined pancreas–endothelium-disabled (*PDX1* and *KDR* KO) pig model [6]. In this case, however, pig fibroblasts were genetically modified with CRISPR/Cas9 in vitro, and SCNT was subsequently carried out to obtain mutated embryos. The advantage of this approach is that, as gene editing is performed on cultured cells, it is possible to select clones with the appropriate gene modification, therefore selecting biallelic site-specific mutations while avoiding mosaicism. Nonetheless, SCNT requires higher technical specialization than CRISPR/Cas9 microinjection, and its efficiency is remarkably lower [10]. In addition, developed pigs often present health complications associated with the process of SCNT and occasionally suffer from sudden death [11,12]. Therefore, the zygote injection of CRISPR/Cas9 is an attractive alternative to SCNT in the production of gene-edited animals as it is more accessible and efficient. 

## 5. Conclusions

In summary, we designed effective gRNAs and optimized their concentration and combination to produce genetically modified porcine embryos via zygote microinjection of the CRISPR/Cas9 system. The results of this study demonstrate that CRISPR/Cas9 zygote editing is a simpler and more efficient process to produce *ETV2* KOs compared to the in vitro gene editing of fibroblasts followed by an SCNT double-step procedure [9]. The large-scale application of this protocol could significantly improve the potential of pig *ETV2* KO embryos as a platform for blastocyst complementation experimentation for the generation of humanized organs in pigs.

## Figures and Tables

**Table 1:** Embryo development after zygote CRISPR/Cas9 injection (gRNA 1, 2, 3, and 4). Zygotes included in the control group were non-injected.

	Putative Zygotes (n)	Cleavage Rates (%)	Blastocyst Rates (%)	Blastocysts Analyzed via NGS
Control	625	306/625 (48.96)	70/306 (22.88)	28
gRNA1	144	86/144 (59.72) *	30/86 (34.88) *	26
gRNA2	72	43/72 (59.72)	26/43 (60.47) *	19
gRNA3	288	145/288 (50.35)	31/145 (21.38)	24
gRNA4	291	141/291 (48.45)	27/141 (19.15)	23

The cleavage rate (%) was calculated as the percentage of cleaved embryos on the total number of putative zygotes used. The blastocyst rate (%) was calculated as the percentage of blastocysts obtained from the total number of cleaved embryos. * *p*-value < 0.05 compared to control.

**Table 2:** Embryo development after zygote injection of CRISPR/Cas9 (combined gRNA 1 and 4) with different RNP component concentrations (5× = 250 ng/μL gRNAs and 500 ng/μL Cas9; 10×= 500 ng/μL gRNAs and 1 μg/μL Cas9). Zygotes included in the control group were non-injected.

	Putative Zygotes (n)	Cleavage Rates (%)	Blastocyst Rates (%)	Blastocysts Analyzed via NGS
Control	48	47/48 (97.92)	21/47 (44.68)	13
gRNA1-4 5×	20	17/20 (85.00) *	9/17 (52.94)	9
gRNA1-4 10×	62	33/62 (53.23) *	10/33 (30.30)	10

The cleavage rate (%) was calculated as the percentage of cleaved embryos on the total number of putative zygotes used. The blastocyst rate (%) was calculated as the percentage of blastocysts obtained from the total number of cleaved embryos. * *p*-value < 0.05 compared to control.

## Data Availability

Not applicable.
